# ABO Blood Group and the Risk of Hepatocellular Carcinoma: A Case-Control Study in Patients with Chronic Hepatitis B

**DOI:** 10.1371/journal.pone.0029928

**Published:** 2012-01-03

**Authors:** Qiang Li, Cui-Hua Yu, Jin-Hong Yu, Li Liu, Shuang-Shuang Xie, Wen-Wen Li, Xia Yang, Wen-Bo Fan, Zhong-Tao Gai, Shi-Jun Chen, Naoya Kato

**Affiliations:** 1 Division of Liver Disease, Jinan Infectious Disease Hospital, Shandong University, Jinan, China; 2 Department of Pharmacy, Jinan Infectious Disease Hospital, Shandong University, Jinan, China; 3 Unit of Disease Control Genome Medicine, Institute of Medical Science, University of Tokyo, Tokyo, Japan; 4 Unit of Hyperbaric Medicine, Shandong Province Hospital, Jinan, China; 5 Unit of Digestive Disease, Department of Internal Medicine, Qihe People's Hospital, Shandong, China; 6 Department of Infectious Disease, Jinan Infectious Disease Hospital, Shandong University, Jinan, China; Broad Institute of Massachusetts Institute of Technology and Harvard University, United States of America

## Abstract

**Background:**

Studies have observed an association between the ABO blood group and risk of certain malignancies. However, no studies of the association with hepatocellular carcinoma (HCC) risk are available. We conducted this hospital-based case-control study to examine the association with HCC in patients with chronic hepatitis B (CHB).

**Methods:**

From January 2004 to December 2008, a total of 6275 consecutive eligible patients with chronic hepatitis B virus (HBV) infection were recruited. 1105 of them were patients with HBV-related HCC and 5,170 patients were CHB without HCC. Multivariate logistic regression models were used to investigate the association between the ABO blood group and HCC risk.

**Results:**

Compared with subjects with blood type O, the adjusted odds ratio (AOR) for the association of those with blood type A and HCC risk was 1.39 [95% confidence interval (CI), 1.05–1.83] after adjusting for age, sex, type 2 diabetes, cirrhosis, hepatitis B e antigen, and HBV DNA. The associations were only statistically significant [AOR (95%CI) = 1.56(1.14–2.13)] for men, for being hepatitis B e antigen positive [AOR (95%CI) = 4.92(2.83–8.57)], for those with cirrhosis [AOR (95%CI), 1.57(1.12–2.20)], and for those with HBV DNA≤10^5^copies/mL [AOR (95%CI), 1.58(1.04–2.42)]. Stratified analysis by sex indicated that compared with those with blood type O, those with blood type B also had a significantly high risk of HCC among men, whereas, those with blood type AB or B had a low risk of HCC among women.

**Conclusions:**

The ABO blood type was associated with the risk of HCC in Chinese patients with CHB. The association was gender-related.

## Introduction

Hepatocellular carcinoma (HCC) is one of the few cancers with well-defined major risk factors, including chronic hepatitis B virus (HBV) and hepatitis C virus (HCV) infection, and cirrhosis due to many causes [1]. However not all patients with these diseases will develop HCC. Therefore within these disease groups, there are other factors that indicate greater or lesser risk[2]. In China, a hyper-endemic area of HBV infection, nearly 80% of HCC cases have been linked to chronic HBV infection, and approximately 60%–90% of these develop in patients with cirrhosis [3], [4]. Older age, male sex, cirrhosis and sustained activity of liver disease are important predicators for HCC[5]. In patients with chronic HBV infection, HBV DNA level, viral genotype, and hepatitis B e antigen (HBeAg) status have been identified as risk factors for HCC [6], [7], [8]. Other potential risk factors, such as diabetes mellitus, alcohol abuse, obesity, and family history of liver cancer may also play a role in the development of HCC [2], [9]. As for human genetic factors, polymorphisms of tumor necrosis factor-α (TNF-α) and epidermal growth factor (EGF) gene have been reported to be associated with HCC risk [10], [11], [12].

The ABO blood group has been previously found to be associated with the risk of several malignancies, including gastric cancer, pancreatic cancer, epithelial ovarian and skin cancer[13]. However, there is little data about the association with HCC risk. Two recent genome-wide association studies (GWAS) suggest that the ABO blood group antigen may affect the systemic inflammatory state. Single nucleotide polymorphisms (SNPs) at the ABO locus were associated with two serum markers of inflammation: TNF-α and soluble intercellular adhesion molecule 1 (sICAM-1) [14], [15]. Enhanced expression of TNF-α is associated with liver inflammation and hepatocarcinogenesis[16], also plasma levels of sICAM-1 are not only associated with the risk of incident diabetes and liver disease activity, known predisposing factors for HCC, but furthermore are also a predictive marker of HCC occurrence and prognosis [17], [18], [19], [20], [21], [22]. These indicate a possible link between the ABO blood group and HCC risk, especially in the presence of other etiological risks for HCC. We hypothesized that certain serotypes of the ABO blood group might provide additional risk of HCC development in the presence of CHB. So, the aim of this case-control study was to examine the association between the ABO blood group and HCC risk among patients with CHB.

## Materials and Methods

### Study population

This case-control study was part of an ongoing hospital-based prospective investigation [23] that was conducted in Jinan Infectious Disease Hospital, Shandong University, a tertiary hospital in Shandong Province, China. The study was approved by the ethics committee of Jinan Infectious Disease Hospital, Shandong University, and written informed consent for participation was obtained from each study participant.

Subjects who fulfilled the following criteria were recruited into the study: hospitalized for HCC or CHB, age ≥30 years, positive for hepatitis B surface antigen (HBsAg), negative for anti-HCV, without a history or other evidence of cancer other than HCC, without a history or other evidence of hepatitis other than hepatitis B, without a history of alcohol consumption, no cancer treatment, and no treatment with nucleotide/nucleosides or interferon, Han population and residence of Shandong Province.

From January 2004 and December 2008, a total of 6,275 consecutive eligible patients (1,105 HBV-related HCC and 5170 CHB without HCC) were enrolled. 882 eligible patients were not recruited because of patient refusal or patient sickness. Statistical analyses indicated that the eligible patients who were not recruited did not differ from the recruited patients in term of demographic and clinical features (retrieved from patients' medical records).

A total of 1105 HBV-related HCC patients were used as the case in the study. The 5170 hospital cross-sectional CHB patients without HCC were used as the control.

### Study variables

The variables analyzed in this study included age, sex, city of residence, family history of liver cancer, type 2 diabetes, hepatitis B e antigen (HBeAg), cirrhosis, HBV DNA level, and the ABO blood group. Biochemical parameters related to impaired liver function, such as, bilirubin, albumin, and prothrombin time, were also analyzed to explore the relationship between the ABO blood group and severity of liver disease.

Upon entry to the hospital, all subjects were interviewed by trained physicians. Demographic data, family histories, and medical histories were collected. Physical examinations, blood counts, the ABO blood type, Rh factor, serum biochemical parameters, prothrombin time, serum alpha-fetoprotein (AFP), anti-HCV (AxSYM HCV, version 3.0, Abbott Laboratories, Abbott Park, IL, USA), hepatitis B surface antigen (HBsAg), anti-HBs, HBeAg, anti-HBe, and anti-HBc (Abbott Laboratories) were all measured. HBV DNA quantification (Roche COBAS HBV Amplicor Monitor assay), ultrasound examinations, and gastrointestinal barium meal X-ray examinations were also performed.

First-degree relatives (parents, siblings, and children) with liver cancer were considered to have a positive family history of liver cancer. Subjects with a fasting blood sugar level of at least 7.0 mmol/L on at least two occasions or those diagnosed with type 2 diabetes before entering the hospital were defined as having type 2 diabetes. At the hospital, a standardized questionnaire was used to interview the patients about their history of alcohol drinking, including drinking frequency, average monthly intake of each type of alcoholic beverage. We assessed total alcohol intake according to the average ethanol content, by volume, of beer (4–5 percent), wine (grape wine, rice wine 8–12 percent), and liquor(38–60 percent)[24]. Subjects with an ethanol intake of 30 g/d or more for men and 20 g/d or more for women for longer than 10 years were considered to have a positive history of alcohol consumption. A structured questionnaire was used to record the information collected.

### Diagnosis of HCC and Cirrhosis

The criteria for diagnosis of HCC were positive histology or cytology, two image findings showing HCC from different sources [ultrasonography, enhanced computed tomography (CT) scan, or magnetic resonance imaging(MRI)], or serum AFP level greater than 400 ng/mL in combination with one positive image finding. Image diagnosis for HCC was based on the following classic imaging manifestations: early enhancement at the arterial phase and hypoattenuation at the portal venous phase or at the equilibrium phase on contrast-enhanced dynamic CT or MRI, and hyperattenuation on CT during hepatic arteriography and hypoattenuation on CT during arterial portography. Of these 1,105 cases, 298 cases were diagnosed by histological pathology, 364 cases with an enhanced computed tomography scan and magnetic resonance imaging, and 443 cases with a finding of AFP higher than 400 ng/mL in combination with an abnormal finding from an enhanced computed tomography scan or ultrasonography.

Diagnosis of cirrhosis was based on liver biopsy or clinical findings combined with at least one image finding from ultrasonography or a computed tomography scan. In 386 CHB patients with a biopsy, the diagnosis of cirrhosis was based on liver histology according to the criteria of Desmet et al. [25]. In those patients without biopsy specimens, the diagnosis of cirrhosis was based on clinical and morphological criteria, ultrasound or computed tomography, according to standard definitions[26]. These included the presence of clinical manifestations of portal hypertension (e.g., esophageal varices, encephalopathy, or ascites), biochemical abnormalities (e.g., decreased serum albumin and platelets or prolonged prothrombin time), and obvious morphological changes in the liver detected by hepatic imaging (e.g., ultrasonography or computed tomography scan). Minor signs were also clinically noted, such as palmar erythema, spider angioma, and clubbing of the fingers. Ultrasonography was performed by experienced radiologists for every patient upon entry to the hospital. The severity of cirrhosis was evaluated using Child-Pugh score[27].

### Statistical Analysis

Demographic and clinical parameters were evaluated using the chi-squared test for categorical variables, independent-Samples t-test for continuous variables with normal distribution, and the Mann-Whitney U test or Kruskal-Wallis test for continuous variables with skewed distribution. Possible confounding effects among the variables were adjusted using a multivariate logistic regression model, and adjusted odds ratios (AORs) and 95% confidence intervals (CI) were calculated. Effect modifications were evaluated by stratification, statistical interaction was assessed by including main effect variables and their product terms in the logistic regression model. For all tests, *P* values were 2 sided. A *P* value of less than 0.05 was considered significant. All data analyses were performed using SPSS v. 13.0 (SPSS Inc., Chicago, IL, USA).

## Results

### Patient Characteristics

The demographic and clinical characteristics of HCC cases and CHB controls are summarized in **Table 1** **.** As expected, most HCC patients were male (84.7%), HBeAg negative (76.8%), and cirrhotic (68.7%). The prevalence of HBV DNA>10^5^copies/mL were lower among cases than among controls. Cases and controls had a similar distribution of family history of liver cancer and city of residence (Jinan and other cities of Shandong Province). HCC patients were older than controls, the mean age ± standard deviation was 53.8±9.3 years for patients with HCC and 44.9±10.7 years for controls (*P*<0.001).

**Table 1:** Demographic and clinical characteristics of hepatitis B virus-related HCC cases and hospital cross-sectional CHB controls: Univarite analyses.

	Cases	Controls		
Variables	N = 1105 (%)	N = 5170 (%)	Univariate OR (95%CI)	*P*
**Sex**				<0.001
Female	169 (15.3)	1353 (26.2)	1	
Male	936 (84.7)	3817 (73.8)	1.96 (1.65–2.34)	
**Age (years)**				<0.001
30–39	82 (7.4)	2014 (39.0)	1	
40–49	250 (22.6)	1385 (26.8)	4.43 (3.42–5.74)	
50–59	470 (42.5)	1247 (24.1)	9.26 (7.25–11.83)	
≥60	303 (27.4)	524 (10.1)	14.20 (10.93–18.46)	
**City of residence**				0.69
Jinan	464 (42.0)	2137 (41.3)	1	
Other cities	641 (58.0)	3033 (58.7)	1.03 (0.90–1.17)	
**Family history** **of liver cancer**				0.76
No	1030 (93.2)	4832 (93.5)	1	
Yes	75 (6.8)	338 (6.5)	1.04 (0.80–1.35)	
**Type 2 diabetes**				0.013
No	1012 (91.6)	4842 (93.7)	1	
Yes	93 (8.4)	328 (6.3)	1.36 (1.07–1.73)	
**HBeAg status**				<0.001
HBeAg+	256 (23.2)	3144 (60.8)	1	
HBeAg−	849 (76.8)	2026 (39.2)	5.15 (4.43–5.98)	
**Cirrhosis**				<0.001
No	346 (31.3)	3559 (68.8)	1	
Yes	759 (68.7)	1611 (31.2)	4.85 (4.21–1.73)	
**HBVDNA>10^5^copies/mL**				<0.001
No	559(50.6)	1153(22.3)	1	
Yes	546(49.4)	4017(77.7)	0.28 (0.24–0.33)	

HCC, hepatocellular carcinoma; CHB, chronic hepatitis B; OR, odds ratio; CI, confidence interval; HBeAg, hepatitis B e antigen.

### ABO blood group and HCC risk

Distributions of blood types O, A, AB, and B are shown in **Table 2**. The distribution of blood type A was higher among HCC cases than among CHB controls (29.0% vs 24.9%, *P* = 0.013), whereas, the distribution of blood type O, AB, and B was similar between cases and controls. Compared with subjects with blood type O, the unadjusted OR for the association of those with blood type A and HCC risk was 1.31(95% CI, 1.06–1.61). In multivariable logistic regression, the AOR was 1.39(95%CI, 1.05–1.83) after adjusting for age, sex, type 2 diabetes, cirrhosis, HBeAg, and HBV DNA.

**Table 2:** ABO blood type and risk for hepatocellular carcinoma development in patients with chronic hepatitis B.

	Cases	Controls			
ABO type	N = 1105 (%)	N = 5170 (%)	Univariate OR (95%CI)	AOR (95%CI)	*P*
O	277 (25.1)	1462 (28.3)	1(reference)	1(reference)	
A	321 (29.0)	1289 (24.9)	1.31 (1.06–1.61)	1.39 (1.05–1.83)	0.021
AB	132 (11.9)	609 (11.8)	1.13 (0.87–1.48)	0.85 (0.58–1.25)	0.409
B	375 (33.9)	1810 (35.0)	1.09 (0.89–1.32)	1.14 (0.87–1.49)	0.337
A+AB	453 (41.0)	1898 (36.7)	1.25 (1.04–1.52)	1.23 (0.95–1.59)	0.122
B+AB	507 (45.9)	2419 (46.8)	1.10 (0.91–1.32)	1.05 (0.81–1.35)	0.732
A+B+AB	828 (74.9)	3708 (71.7)	1.17 (0.99–1.39)	1.18 (0.93–1.49)	0.175

AOR, adjusted odds ratio; CI, confidence interval.

Logistic regression model adjusted for sex, age, type 2 diabetes, cirrhosis, HBeAg status, and HBVDNA.

We further assessed whether the association between the ABO blood group and HCC risk differed according to strata of other known risk factors for HCC, including sex, HBeAg, HBV DNA level, and cirrhosis. An interaction between ABO blood type and sex or HBeAg was observed. The AOR (95% CI) for the interaction term of blood type A, B, or AB and sex was 1.57 (0.78–3.17), 3.33 (1.60–6.92), and 8.93 (1.91–41.64), respectively. The AOR (95%) for the interaction term of blood type A, B, or AB and HBeAg was 4.82 (2.58–9.02), 2.30 (1.23–4.32), and 2.64 (1.11–6.30), respectively. As shown in **Table 3**, among male patients, compared to subjects with blood type O, those with blood type A, or B were at a greater HCC risk, whereas, among female patients, compared with blood type O, those with blood type AB or B were at a lesser HCC risk. A strong association between the ABO blood group and HCC risk was observed among HBeAg positive patients, but not among HBeAg negative patients.

**Table 3:** ABO blood type and hepatocellular carcinoma risk in patients with chronic hepatitis B: stratified multivariate analyses.

	ABO blood type				
Factors	O	A	AB	B	A+AB+B
**Sex**					
Female					
No. of cases/controls	64/395	47/311	7/130	51/517	105/958
AOR(95%CI)	1 (reference)	1.07(0.56–2.06)	0.11(0.02–0.53)	0.45(0.23–0.88)	0.55(0.32–0.93)
***P*** ** value**		0.83	0.005	0.019	0.027
Male					
No. of cases/controls	213/1067	274/978	125/479	324/1293	723/2750
AOR(95%CI)	1(reference)	1.56(1.14–2.13)	1.15(0.76–1.73)	1.41(1.05–1.91)	1.42(1.09–1.86)
***P*** ** value**		0.005	0.511	0.024	0.009
**HBeAg status**					
HBeAg positive					
No. of cases/controls	47/927	100/708	19/377	90/1132	209/2217
AOR(95%CI)	1(reference)	4.92(2.83–8.57)	1.89(0.87–4.12)	2.25(1.28–3.96)	3.03(1.83–5.03)
***P*** ** value**		<0.001	0.11	0.005	<0.001
HBeAg negative					
No. of cases/controls	230/535	221/581	113/232	285/678	619/1491
AOR(95%CI)	1(reference)	0.81(0.58–1.12)	0.63(0.41–0.99)	0.90(0.66–1.23)	0.82(0.62–1.07)
***P*** ** value**		0.203	0.043	0.519	0.15
**Cirrhosis**					
No					
No. of cases/controls	100/995	105/866	41/432	100/1266	246/2564
AOR(95%CI)	1(reference)	1.10(0.67–1.79)	0.77(0.40–1.48)	0.85(0.52–1.41)	0.92(0.60–1.40)
***P*** ** value**		0.803	0.442	0.515	0.69
Yes					
No. of cases/controls	177/467	216/423	91/177	275/544	582/1144
AOR(95%CI)	1(reference)	1.57(1.12–2.20)	0.86(0.54–1.38)	1.32(0.96–1.81)	1.32(1.0–1.75)
***P*** ** value**		0.008	0.537	0.090	0.05
**HBV DNA>10^5^copies/mL**					
No					
No. of cases/controls	125/354	143/306	78/136	203/357	424/799
AOR(95%CI)	1(reference)	1.58(1.04–2.42)	1.08(0.63–1.84)	1.68(1.13–2.50)	1.52(1.07–2.17)
***P*** ** value**		0.033	0.776	0.011	0.02
Yes					
No. of cases/controls	152/1108	168/983	54/473	172/1453	394/2909
AOR(95%CI)	1(reference)	1.22(0.85–1.77)	0.71(0.41–1.25)	0.80(0.56–1.16)	0.94(0.68–1.29)
***P*** ** value**		0.282	0.234	0.239	0.69

AOR, adjusted odds ratio; CI, confidence interval; HBeAg, hepatitis B e antigen. Logistic regression model adjusted for sex, age, type 2 diabetes, cirrhosis, HBeAg status, and HBVDNA, and excluding the stratification variable.

### ABO blood group and severity of liver disease

As shown in **Table 4**, subjects with blood type A had more severely impaired liver function. Compared with those with other blood types, subjects with blood type A had a significantly high prevalence of abnormal bilirubin and prolonged prothrombin time. The distribution of cirrhosis was not significantly higher among subjects with blood type A than among those with other blood types. However, among the age<55 years group, the prevalence of cirrhosis was significantly higher among subjects with blood type A than among those with non-A. This indicated a relationship between blood type A and early onset of cirrhosis.

**Table 4:** ABO Blood type and severity of liver disease in chronic Hepatitis B.

	O	A	AB	B	Non-A		
Variables	N (%)	N (%)	N (%)	N (%)	N (%)	*P* #	*P* *
							
Bilirubin≥50 µmol/L	314 (28.7)	322 (33.2)	113 (23.9)	327 (23.9)	754 (25.7)	<0.001	<0.001
Albumin<35g/L	317 (28.9)	305 (31.4)	143 (30.2)	365 (26.7)	825 (28.1)	0.125	0.065
Prothrombin≥18.5s	147 (13.4)	157 (16.2)	69 (14.6)	149 (10.9)	365 (12.4)	0.011	0.011
**With Cirrhosis**	644 (37.0)	639 (39.7)	268 (36.1)	819 (37.5)	1731 (37.1)	0.339	0.082
Age<55years	398 (30.8)	430 (35.2)	166 (29.9)	493 (29.9)	1057 (30.2)	0.032	0.004
Age≥55years	246 (55.3)	209 (53.7)	102 (54.8)	326 (60.5)	674 (57.6)	0.314	0.304

# Chi-Square test among blood type O, A, AB, and B;

*A vs. Non-A.

## Discussion

In this large case-control study in Chinese patients with CHB, we observed a significantly elevated risk for HCC development among those having blood type A, compared to those having blood type O. Stratified analysis indicated that the association was significant in men and not significant in women after adjusting confounding factors. Compared with men with blood type O, those with blood type A or B had a significantly high HCC risk that was independent of established HCC risk factors. To the best of our knowledge, this is the first study showing an association between the ABO blood group and HCC risk in patients with CHB. However, a few previous studies showed relationships between the ABO blood group and liver diseases, including HCC [28], [29], [30], [31], so the discovery of a relationship between the ABO blood group and HCC is not surprising.

There are three reasons. First, the ABO blood group was associated with the severity of liver disease, one of the major predicators for HCC[5]. Poujol-Robert et al. reported that non-O blood group is an independent risk factor for the progression of liver fibrosis in HCV infection[28]. We also observed the association between the ABO blood group and severity of liver disease in our study population. Compared to those with blood type O, those with blood type A had a significantly more severely impaired liver function and an earlier onset of cirrhosis. These indicated an association between the ABO blood group and liver inflammation and fibrosis progression in patients with CHB.

The second reason that a relationship between the ABO blood group and HCC is that the ABO blood group in conjunction with several important cytokines known to be related to HCC development, including EGF, TNF-α, sICAM-1, E-selectin, and P-selectin et al [14], [15], [32], [33], [34].

The EGF receptor (EGFR) plays a link between inflammation and liver cancer[35], and EGF gene polymorphisms have been reported to be associated with HCC risk[10], [12]. The binding of epidermal growth EGF to EGFR is different in blood group A and O, and loss or gain of expression of the A antigen may significantly alter the binding properties of that protein affecting cell signaling and growth[33]. An increase in the number of high affinity EGF binding sites was observed in donors with blood group A1-erythrocytes as compared to red cells taken from donors with blood groups O and B. Glycosyltranferase specificity has broad implications, beyond defining the ABO blood group. Glycoconjugates are important mediators of intercellular adhesion and membrane signaling, two processes integral to malignant progression and spread [36]. The results of two recent GWAS which reported the associations of SNPs at the ABO locus with plasma levels of TNF-α and sICAM-1 also suggested a possible link between the ABO blood group and HCC risk [14], [15].

TNF is a pleiotropic cytokine involved not only in apoptosis, but also with inflammation, hepatocyte protection, proliferation and hepatocarcinogenesis [10], [37]. Polymorphisms of TNF-α is associated with HCC risk [11].The mechanism of the association between the ABO blood group and TNF-α levels is not known. Potentially, the TNF-α-ABO association is mechanistically related to the association between E-selectin and ABO because it is known that TNF-α induces E-selectin expression and is positively associated with E-selectin levels[34], [38], [39]. Interestingly, another GWAS reported the ABO is a major locus for serum soluble E-selectin levels[34]. Whether the association between levels of TNF-α and E-selectin can be explained by their respective association with ABO is not clear.

ICAM-1 belongs to the immunoglobulin superfamily and plays an important role in the regulation of immune response, particularly in the antigen-presenting mechanism[40]. Plasma levels of sICAM-1 have been reported to be associated with liver disease activity, HCC occurrence and prognosis [17], [20], [21], [22]. There is growing evidence that the ABO blood group is highly significantly associated with variation in the levels of a number of biomarkers. A very recent large-scale genomic study revealed sP-selectin levels were also associated with ABO gene variants and the association was accounted for by the A1 allele of the ABO blood group [32].

P-selectin, which has important roles in inflammatory processes, tumor formation and metastasis, is a member of the selectin family of adhesion molecules and is expressed mainly at the surface of platelets and endothelial cells [41], [42].These indicated a possible association of ABO blood group with platelets. Platelets are key actors in inflammation and critical for liver regeneration[43]. Interestingly, an association between the ABO blood group and thrombocytopenia in patients with CHB was observed (unpublished data). Thrombocytopenia has been regarded as a surrogate of liver fibrosis and a predicator of HCC [44], [45]. So, platelets might mediate another possible inflammatory pathway connecting the ABO blood group with liver fibrosis and HCC risk.

The physiological role of the ABO blood group still remains enigmatic. The ABO blood type loci are not in linkage with genes encoding ICAM, E-selectin, and P-selectin. ABO gene product related glycosylation might influence sheding/cleavage of these biomarkers from the endothelium, probably by glycosylation of P-selectin, E-selectin, and ICAM-1[46]. Glycosylation could also affect the clearance rate of sP-selectin, sE-selectin, and SICAM-1 from blood[32], [34]. Decreased cleavage of adhesion molecules from endothelial cells associated with A allele would mean more adhesion molecules on the endothelial cells, increased adhesion and inflammation[14]. In conclusion, collective data suggested that the ABO blood type A might increase HCC risk by connecting with several inflammatory pathways.

Third, abnormal expression ABO blood antigens in liver tissue might be another possible explanation for a relationship between the ABO blood group and HCC. ABO blood antigens (A, B, H) usually express on the surface of red blood cells and most epithelial tissue, but not on hepatocytes, sinusoidal endothelial cells, and bile canaliculi of the normal liver[47]. However, an increased ABH expression or neoexpression was observed in HCC tissues. Terada et al. reported that expression of the ABO blood group antigen was more severe in atypical adenomatous hyperplasia and hepatocellular carcinoma than in normal liver and chronic hepatitis[30]. Okada found neoexpression of ABH blood group antigens in HCC tissues[31]. Expression of H-active glycolipid was enhanced in HCC tissues from the patients with blood types other than O. These suggest that alterations in glycosyltransferase specificity may occur during hepatocarcinogenesis. Recently, Hoshida and colleagues provided new insights into genome-based predictors of outcome in HCC patients [48].If abnormal expression of the ABO blood group antigen in liver tissue could predict HCC occurrence and outcome warrants further study.

We observed a gender difference in the association between the ABO blood group and HCC risk, women with blood group AB or B had a significantly lower HCC risk than those with blood group O. Because of the small number of women with blood type AB in the present study, the result should be considered with caution. The underlying mechanism of the gender difference is unknown. However, gender-related HCC risk difference is universal. The overall male to female ratio of HCC is about 2∶1 to 4∶1[3]. A gender difference was observed in that interleukin-6 signaling promotes chemically induced HCC in genetic mouse models [49]. Several epidemiological studies also showed gender-based dimorphism for the association between other risk factors and HCC development [50], [51]. The present data indicated blood type A was an additional HCC risk in the presence of other potent HCC etiological risk factors. However, in women, a low HCC risk population compared with men, the role of blood A antigen on HCC development might be not potent enough to become a significant additional HCC risk factor. Interestingly, another finding that there was no significant association between blood type A and HCC risk among subjects without cirrhosis, a low HCC risk population compared with those with cirrhosis, also supported the notion.

This study has several notable strengths. First its large sample size enabled us to get a meaningful finding of the association between the ABO blood group and HCC risk in patients with CHB. Second, inpatient CHB controls in this study largely represents the population from which the HBV-related HCC cases arose. In areas of high HBV endemicity, persons with cirrhosis have an approximately 3-fold higher risk for HCC than those with chronic hepatitis but without cirrhosis and a 16-fold higher HCC risk than the inactive carrier[5]. So, most HBV-related HCC arose from CHB with or without cirrhosis, but not from an inactive HBsAg carrier. Third, some possible confounding factors, such as cirrhosis status, alcohol consumption, family history of liver cancer, city of residence, HBeAg status, and HBV DNA were evaluated in this study.

There are certain limitations to the study. First, the study population was composed primarily of Chinese Han patients with CHB, which somewhat limits the generalization of the results. Further studies are necessary to confirm the association in other population and in patients with other underlying liver diseases. Second, not all HCC and cirrhosis cases were histologically diagnosed. However, regarding HCC diagnosis, all patients with nodules of 1–2 cm were confirmed with positive cytohistological findings by ultrasound-guided liver biopsy. All of the patients were re-evaluated during the hospitalization period. Moreover, hospitalized HCC patients in this study were followed-up every 1–3 months in the outpatient division of our hospital. Thus, it is believed that there were no cases of misdiagnosed HCC, or, at the very least, there were very few.

In conclusion, the results suggested that ABO blood type is associated with the risk of HCC in Chinese patients with CHB. The ABO blood group A increased HCC risk in patients with CHB independent of other major HCC risk factors, and this association was gender-related. Further studies are necessary to confirm the association in patients with other underlying liver diseases and to elaborate mechanisms by which ABO antigens may influence HCC risk. In the future, it is possible that the ABO blood type could be incorporated into predictive models for HBV-related HCC, together with other human genetic, HBV-related risk, and environmental factors.
